# A machine learning-based predictive model for the occurrence of lower extremity deep vein thrombosis after laparoscopic surgery in abdominal surgery

**DOI:** 10.3389/fsurg.2025.1502944

**Published:** 2025-05-30

**Authors:** Su-Zhen Yang, Ming-Hui Peng, Quan Lin, Shi-Wei Guan, Kai-Lun Zhang, Hai-Bo Yu

**Affiliations:** Department of Hepatobiliary Surgery, Wenzhou Central Hospital, Dingli Clinical Institute of Wenzhou Medical University, Wenzhou, Zhejiang, China

**Keywords:** artificial intelligence, nursing diagnosis, postoperative care, clinical supervision, laparoscopic surgery, deep vein thrombosis

## Abstract

**Background & aims:**

Deep vein thrombosis, a common complication after laparoscopic surgery, can negatively affect patients' limb motor function and even seriously threaten their lives. Therefore, it is crucial to accurately identify patients at high risk of lower extremity deep vein thrombosis. Thus, the aim of this study was to develop a model to predict the occurrence of deep vein thrombosis in patients after laparoscopy.

**Methods:**

We retrospectively analyzed the clinical data of patients who underwent laparoscopic surgery at Wenzhou Central Hospital's Hepatobiliary Surgery Department. Patients with postoperative deep vein thrombosis composed the observation group, while others composed the control group. Eleven key features were identified through group comparisons and used for model development. Twenty machine learning algorithms were evaluated, and the top five algorithms were used to build the final model by stacking.

**Results:**

A total of 335 patients underwent laparoscopic abdominal surgery. Patients with deep vein thrombosis (9.9%) differed significantly in age, history of tumor, hemoglobin, red blood cell counts, preoperative blood pressure, duration of the surgery, activated partial thromboplastin time, D-dimer, total protein, albumin, and calcium. According to our model, the most important features influencing the predictions were tumor history, age, time to surgery, and D-dimer level. We employed two interpretability methods: decomposition interpretation and Shapley additive explanation. Decomposition analysis revealed that the three study characteristics with the strongest predictive effect for deep vein thrombosis occurrence after laparoscopy were, in descending order, the time of surgery, patient age, and tumor history. Conversely, for ruling out deep vein thrombosis, the most important features were tumor history, hemoglobin level, and age. Shapley additive explanation revealed that tumor history, age, and time of surgery were the most important factors for predicting and ruling out deep vein thrombosis following laparoscopy. We additionally selected 114 patients for external validation, and the results showed that the ROC of validation set for the LASDVT model was 0.9293 and the AUPRC was 0.6497. The effect of the LASDVT model was statistically different (delong test, *p* = 0.0047) and superior to the Caprini score.

**Conclusion:**

We present a model for predicting deep vein thrombosis in laparoscopic surgery patients. This model outperformed the Caprini score in predicting the incidence of deep vein thrombosis.

## Introduction

As the global population ages at an accelerated pace, the elderly are increasingly becoming major consumers of healthcare services. This demographic shift presents both challenges and opportunities for the advancement of medical technology, particularly in the field of minimally invasive surgery. Traditional abdominal surgeries typically involve large incisions, leading to significant postoperative pain and prolonged recovery times for patients. However, with the continuous evolution of medical technology, especially the maturation of laparoscopic techniques, minimally invasive surgery has gradually supplanted traditional open surgery, establishing itself as the predominant approach in abdominal surgery. Minimally invasive surgical techniques have steadily supplanted past open surgical interventions for the treatment of benign and malignant diseases in abdominal surgery (1–5). Minimally invasive techniques offer significant clinical benefits to most patients. These benefits include a faster return to normal function, a shorter hospital stay, and a reduced risk of complications. As medical technology advances, minimally invasive surgical techniques are continually being refined. Alongside traditional laparoscopy, innovative approaches like single-port laparoscopic surgery and natural orifice transluminal endoscopic surgery (NOTES) are emerging. These newer methods further minimize surgical trauma and enhance patient safety. Conversely, open surgery carries the drawbacks of increased invasiveness, leading to higher blood loss, more postoperative pain, extended hospital stays, and larger incisions. Consequently, minimally invasive surgery has become the preferred approach in contemporary abdominal surgery (6, 7). Nurses play a crucial role in minimally invasive procedures, from preoperative education and postoperative care to managing nutrition and pain relief (8). Although minimally invasive techniques are the gold standard in many surgical fields, the complications of laparoscopic surgery are just as serious (9). Examples include injury to adjacent organs, intraoperative and postoperative bleeding, gastrointestinal fistulae, incisions and abdominal infections, postoperative abdominal or abdominal wall implantation of tumors and postoperative deep vein thrombosis of the lower extremities. Given the crucial role that clinicians and nurses play in identifying patient complications, ongoing development of clinical techniques is essential to enhance their ability to swiftly recognize complications associated with minimally invasive surgery.

Deep vein thrombosis (DVT), a common complication after laparoscopic surgery, usually occurs within one week after surgery. DVT can significantly impair patients' limb motor function, leading to a decreased quality of life. Furthermore, detachment of a venous thrombus can trigger acute pulmonary embolism, a life-threatening condition for patients (10–12). Studies have demonstrated that minimally invasive surgery may have a lower overall risk of venous thromboembolism (VTE) compared to open surgery (13–15). However, some research suggests a higher incidence of postoperative deep vein thrombosis (DVT) specifically after laparoscopic procedures. Regardless of the surgical approach (open or minimally invasive), VTE remains a potential complication. Importantly, the occurrence of VTE in any patient, irrespective of the surgical method, can have serious adverse consequences, significantly impacting their life and quality of survival. Therefore, accurately identifying patients at risk for lower extremity deep vein thrombosis (LEDVT) is crucial (16, 17). Currently, the diagnosis and prediction of lower extremity deep vein thrombosis (LEDVT) primarily rely on scoring systems such as the Caprini and Padua scores, along with laboratory tests and ultrasonography (18). These scoring systems were originally published in the 1990s and early 2000s and were found to be valuable for predicting the occurrence of LEDVT after open surgery. However, their ability to accurately predict outcomes in laparoscopic surgery remains unclear (19, 20). There are some deficiencies in Caprini scores. For example, the Caprini score's classification of risk classes is based on data from Western populations and may not be applicable to other populations, especially those at high risk for VTE, such as oncology patients and orthopedic patients. Moreover, the risk factor assignment of the Caprini score is based on the relative contribution of different factors to the risk of VTE, but there may be interactions or synergistic effects between these factors, and the Caprini score does not consider the combined effects of these factors (21).

In summary, the diagnosis and prediction of DVT, a common complication after laparoscopic surgery, relies on the Caprini score and Padua score, as well as laboratory tests and ultrasonography, but each of these methods has its own drawbacks. This study aimed to identify independent risk factors for laparoscopic abdominal surgery patients who develop LEDVT. By doing so, we hope to develop a more accurate and efficient prediction model to aid clinicians and nurses in accelerating patient recovery.

## Materials and methods

### Institutional review board approval

This retrospective study was approved by the Institutional Review Board of Wenzhou Central Hospital (202402192124000497228).

### Ethical compliance with human study

This study was conducted in compliance with the ethical standards of the responsible institution on human subjects as well as with the Helsinki Declaration.

### Study design and cohort

This research was guided by the Declaration of Helsinki. The clinical charts and related data of patients who underwent laparoscopic surgery from January 3, 2023, to December 19, 2023, in our hepatobiliary surgery department were collected and reviewed. Patients who met the following criteria were included in this study: (1) underwent laparoscopic surgery; (2) complete clinical data; (3) no preoperative use of hormonal drugs; (4) Patients who did not undergo secondary surgery. Patients who met any of the following criteria were excluded: (1) incomplete clinical data; (2) severe preoperative underlying disease or intolerance to general anesthesia; (3) underwent surgery via open surgery or other nonlaparoscopic surgery; (4) were perinatal women. A total of 335 patients were included in the study. Among these patients, 33 developed LEDVT postoperatively and composed the observation group. The remaining 302 patients who did not develop LEDVT after laparoscopy composed the control group. We additionally selected 114 patients who underwent laparoscopic surgery at our hospital from December 2023 to August 2024 for external validation, with the same inclusion criteria as above.

### Data collection and variables

The clinical records and data of the patients were collected. The diagnostic criteria for LEDVT are that the vascular ultrasound must show hypoechoicity, loss of blood flow signal in the lumen of the vessel, and no change after applying pressure to the vessel with the probe. The study variables included age, gender, tumor history, height, weight, preoperative blood pressure, duration of surgery, prothrombin percentage activity, hemoglobin, red blood cell count, prothrombin time (PT), fibrinogen, thrombin time (TT), activated partial thromboplastin time (APTT), D-dimer, white blood cell count, absolute neutrophil count, absolute lymphocyte count, mean corpuscular hemoglobin, platelet count, total protein, albumin, globulin, total bilirubin, direct bilirubin, indirect bilirubin, glucose, urea, calcium, and C-reactive protein (CRP).

### Statistical analysis

**Figure 1:**
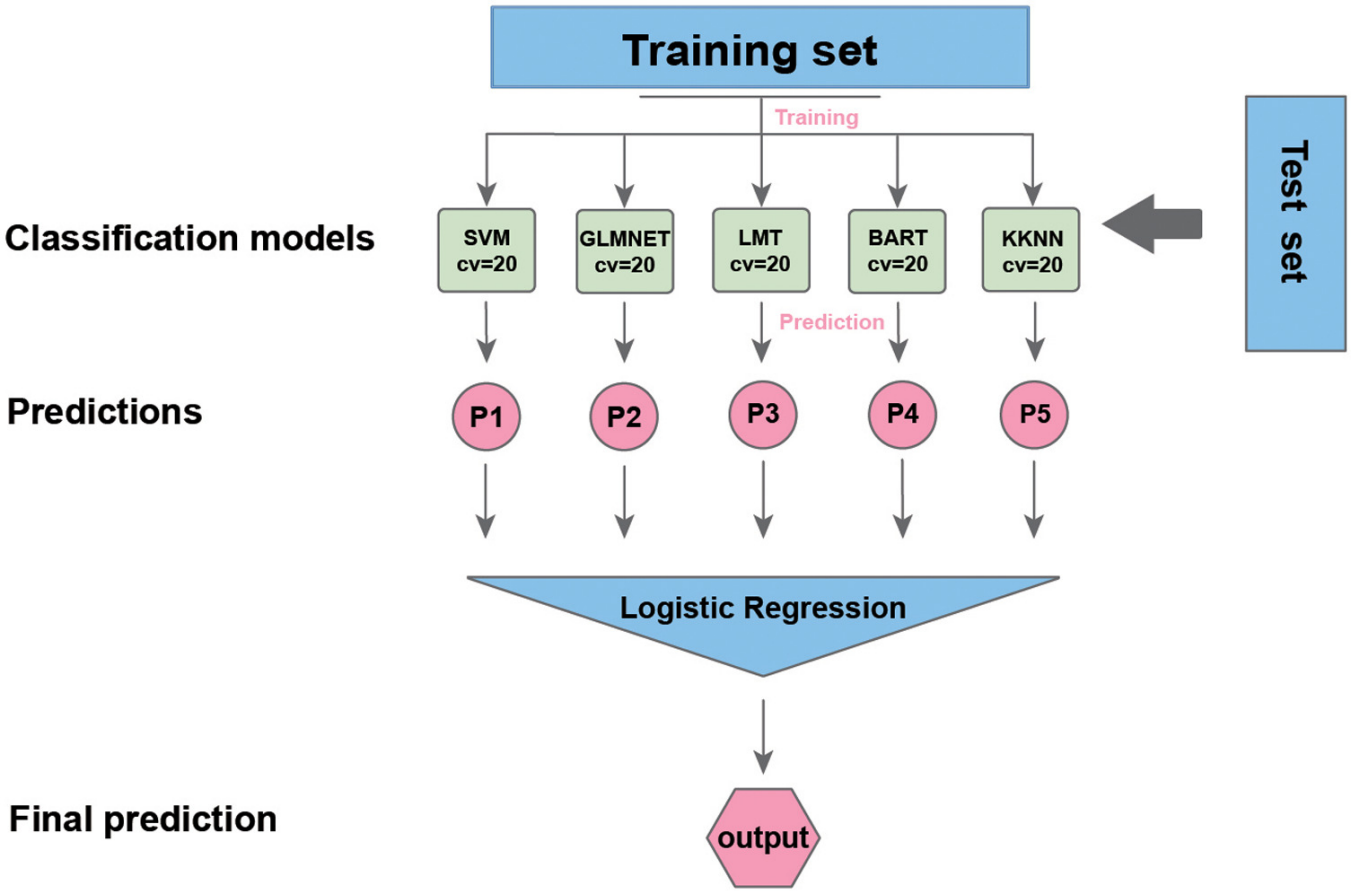
The workflow of our study. SVM, support vector machine; LMT, locally mapped trees; BART, Bayesian additive regression trees; KKNN, kernel K-nearest neighbors.

The data were analyzed using R (version 4.2.2, https://www.r-project.org/). Categorical variables were compared with the chi-squared test. Student's *t* test was used to compare continuous variables between two groups when the data were normally distributed and had homogeneous variance. The data are presented as the median (P25, P75), and the nonparametric rank sum test was used when the measurement data did not have a normal distribution or homogeneity of variance. We constructed a laparoscopic abdominal surgery deep vein thrombosis (LASDVT) model. The construction of the machine learning (ML) model is based on the mlr3 package, benchmarking of extracted meaningful features using 20 algorithms, and evaluation by ML evaluation metrics such as the ACC, AUROC, AUPRC, classification error (CE), F1 score, false discovery rate (FDR), false negative rate (FNR), false positive rate (FPR), precision, true positive rate (TPR), and true negative rate (TNR) using 5-fold cross-validation with 200 repetitions. The five algorithms with the best overall evaluation were selected to construct the final model using the starking approach. We divided the dataset into training and test sets at a ratio of 7:3. The class imbalance in the training set consisted of 26 cases (10.7%), while the test set had 7 cases (11.6%) of class imbalance. The LASDVT model is trained on the training set, and the performance of the model is validated on the test set. The LASDVT model interpretation was performed using the DALEX R package (Figure 1). A *p*-value of <0.05 was considered to indicate statistical significance.

### Feature importance analysis

We use two interpretable methods to determine the importance of features in machine learning models: global and local interpretation.

For global interpretation, we use the Predictor class of the iml package to construct the prediction interpreter object. The feature importance calculation is based on the cross-entropy loss function (cross-entropy loss), which quantifies the contribution of each feature to the predictive power of the model by performing 100 repetitive samples through the replacement eigenvalue method. Specifically, by perturbing the values of each feature and observing the changes in model performance, a feature importance score is calculated, with higher scores indicating that the feature has a greater impact on model prediction. The model-independence of PFI makes it suitable for stacked integration models, avoiding algorithmic idiosyncratic biases based on the number of tree model splits, etc.; at the same time, combining it with univariate analysis mitigates the underestimation of importance of multi-covariate features.

In terms of local interpretation, to assess the importance of local features, we used the iBreakDown package and Shapley Additive Explanations (SHAP) within the mlr3 framework. The feature contributions of one randomly selected patient without lower limb thrombosis and one with lower limb thrombosis were quantified using the break_down function, while SHAP values were calculated to assess marginal feature impact.

This dual approach ensured reliable and interpretable insight into the model decision-making process.

## Results

### Participant characteristics and univariate analysis

**Figure 2:**
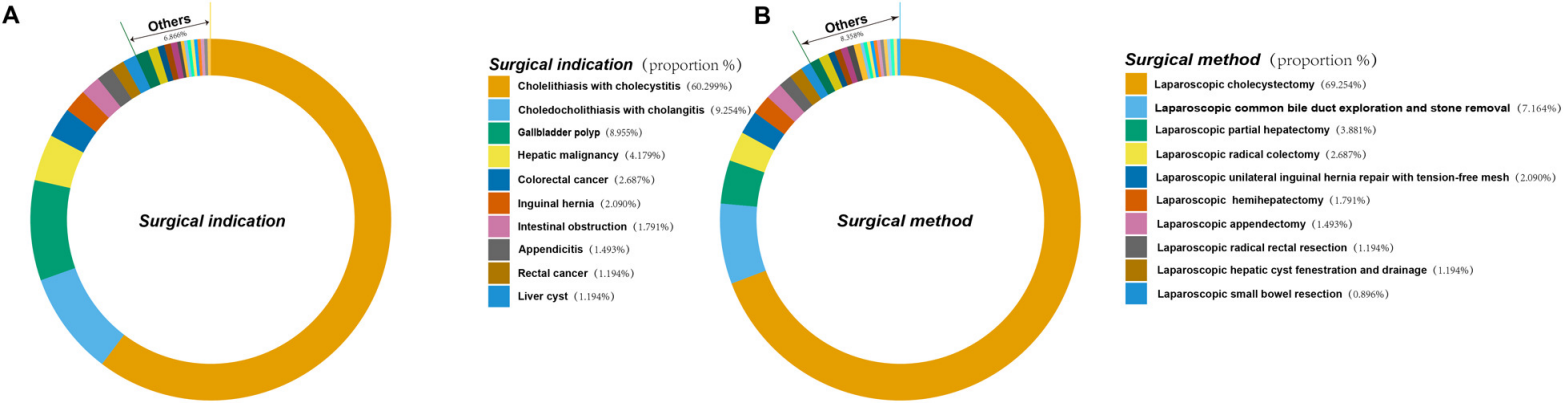
Indications and types of surgery and their percentage in 335 patients who underwent laparoscopic surgery. **(A)** Indications for surgery and their percentage in 335 patients undergoing laparoscopic surgery. **(B)** Type of surgery and its percentage in 335 patients who underwent laparoscopic surgery.

The clinical characteristics of the 335 patients are summarized in Table 1. Figure 2 illustrates the surgical indications, types of surgery, and their respective percentages among 335 patients who underwent laparoscopic procedures. This cohort encompassed a diverse spectrum of surgical indications, encompassing most common laparoscopic procedures. Hepatobiliary and pancreatic surgeries were most frequent, with patients presenting with gallstones and cholecystitis comprising the largest proportion (60.299%) (Figure 2A). Concurrently, laparoscopic cholecystectomy emerged as the most prevalent surgical intervention within this study population, accounting for 69.254% of all procedures (Figure 2B). Univariate analysis revealed no statistically significant differences between the two groups in terms of gender, prothrombin percentage activity, body weight, height, PT, fibrinogen, TT, white blood cell count, absolute neutrophil count, absolute lymphocyte count, mean corpuscular hemoglobin, platelet count, globulin, total bilirubin, direct bilirubin, indirect bilirubin, glucose, urea, or C-reactive protein (*P* > 0.05; Table 2). However, age, tumor history, hemoglobin, red blood cell count, preoperative blood pressure, duration of surgery, APTT, D-dimer, total protein, albumin, and calcium were found to be significantly different between the two groups (*P* < 0.05; Table 2). The mean age of patients who developed LEDVT after laparoscopy was significantly higher compared to those who did not develop LEDVT after surgery (70.788 ± 10.939 vs. 56.563 ± 14.828, *P* < 0.05). Furthermore, the surgical duration was significantly longer in the group that subsequently developed LEDVT compared to the group that did not experience LEDVT postoperatively. [175 (120,287) vs. 60 (50.25, 98.75), *P* < 0.05]. The proportion of patients with a history of tumors was much higher in the group that developed LEDVT than in the group that did not develop LEDVT (*P* < 0.05; Table 2). To build our ML model, we considered only features that showed statistically significant differences (*p* < 0.05) between the groups being compared.

**Table 1:** Baseline information for 335 patients.

Characteristics	Parameter (*n* = 335)
Age (years)	57.964 ± 15.088
Gender	
Male	167 (49.85%)
Female	168 (50.15%)
Tumor history	
Yes	36 (10.75%)
No	299 (89.25%)
Prothrombin percentage activity(%)	103.675 ± 14.244
Hemoglobin(g/L)	130.099 ± 18.275
Red blood cell count(10^12^/L)	4.371 ± 0.618
Body weight(kg)	62 (57,70)
Height(cm)	162 (156,170)
Preoperative blood pressure(mmHg)	127 (115,140.5)
Duration of surgery(min)	67 (53.5,120)
Prothrombin time(s)	11.2 (10.7,12)
Fibrinogen(g/L)	3.29 (2.89,3.96)
Thrombin time(s)	15 (14.3,15.8)
Activated partial thromboplastin time(s)	30.4 (28.6,32.5)
D-dimer(µg/L)	143 (137,301.5)
White blood cell count(10^9^/L)	6.2 (5.1,7.9)
Absolute neutrophil count(10^9^/L)	3.6 (2.9,5.25)
Absolute lymphocyte count(10^9^/L)	1.7 (1.3,2.1)
mean corpuscular hemoglobin (pg)	29.9 (28.9,30.9)
Platelet count(10^9^/L)	219 (181.5,258.5)
Total protein(g/L)	67.4 (63,71.9)
Albumin(g/L)	39.6 (36.4,43.5)
Globulin(g/L)	27.9 (25.5,30.1)
Total bilirubin(µmol/L)	13.7 (10.8,19.9)
Direct bilirubin(µmol/L)	2.7 (2.1,4.15)
Indirect bilirubin(µmol/L)	10.9 (8.5,15.4)
Glucose(mmol/L)	5.4 (4.8,6.2)
Urea(mmol/L)	5 (4.2,6)
Calcium(mmol/L)	2.27 (2.17,2.33)
C-reactive protein(mg/L)	3 (1.3,15.4)
DVT	
Yes	33(9.9%)
No	302(90.1%)

**Table 2:** An intergroup comparison of 335 patients.

Parameter	DVT (*n* = 33)	without DVT (*n* = 302)	*p*-value
Age	70.788 ± 10.939	56.563 ± 14.828	1.73e-08
Gender			
Male	14 (42.42%)	153 (50.66%)	0.4744
Female	19 (57.58%)	149 (49.34%)	
Tumor history			
YES	17 (51.51%)	19 (6.29%)	1.739e-14
NO	16 (48.48%)	283 (93.71%)	
Prothrombin percentage activity(%)	99.121 ± 15.562	104.172 ± 14.031	8.20e-02
Hemoglobin(g/L)	117.303 ± 21.09	131.497 ± 17.418	6.42e-04
Red blood cell count(10^12^/L)	3.84 ± 0.602	4.429 ± 0.593	4.23e-06
Body weight(kg)	60 (57,67)	62 (58,70)	5.88e-01
Height(cm)	158 (155,168)	162 (157,170)	1.31e-01
Preoperative blood pressure(mmHg)	138 (115,149)	125.5 (115,139.75)	1.94e-02
Duration of surgery(min)	175 (120,287)	60 (50.25,98.75)	1.19e-08
Prothrombin time(s)	11.2 (10.9,12.4)	11.2 (10.7,11.9)	3.20e-01
Fibrinogen(g/L)	3.2 (2.88,4)	3.3 (2.89,3.95)	9.86e-01
Thrombin time(s)	15.2 (14.2,15.9)	15 (14.3,15.7)	5.80e-01
Activated partial thromboplastin time(s)	28.9 (27.3,30.3)	30.6 (28.7,32.9)	1.70e-03
D-dimer( *μ* g/L)	326 (183,1944)	137 (137,262.75)	9.91e-06
White blood cell count(10^9^/L)	6.2 (5,8.1)	6.3 (5.1,7.9)	8.47e-01
Absolute neutrophil count(10^9^/L)	3.9 (3,5.9)	3.6 (2.9,5.2)	3.70e-01
Absolute lymphocyte count(10^9^/L)	1.5 (1,1.9)	1.7 (1.3,2.1)	8.42e-02
mean corpuscular hemoglobin (pg)	30.3 (29.4,31.4)	29.9 (28.9,30.9)	1.82e-01
Platelet count(10^9^/L)	203 (164,241)	220 (183,260.5)	2.42e-01
Total protein(g/L)	63.7 (60.7,66.5)	68.1 (63.475,72.575)	2.26e-05
Albumin(g/L)	36.4 (32.6,39.5)	40.1 (37.025,43.775)	2.70e-06
Globulin(g/L)	28.5 (24.6,29.6)	27.85 (25.625,30.1)	3.91e-01
Total bilirubin(µmol/L)	12.9 (8.7,19.8)	13.7 (10.9,19.9)	4.74e-01
Direct bilirubin(μmol/L)	3.1 (2.1,4.2)	2.7 (2,4.1)	5.94e-01
Indirect bilirubin(μmol/L)	10 (7.3,16)	10.9 (8.5,15.325)	4.30e-01
Glucose(mmol/L)	5.6 (4.9,6.6)	5.3 (4.8,6.2)	1.54e-01
Urea(mmol/L)	5.1 (4.3,6.6)	5 (4.2,5.9)	5.38e-01
Calcium(mmol/L)	2.17 (2.11,2.29)	2.27 (2.183,2.34)	1.85e-03
C-reactive protein(mg/L)	6(1.9,22.7)	2.8(1.3,13.875)	1.56e-01

DVT, deep venous thrombosis.

### Construction of the ML model

**Figure 3:**
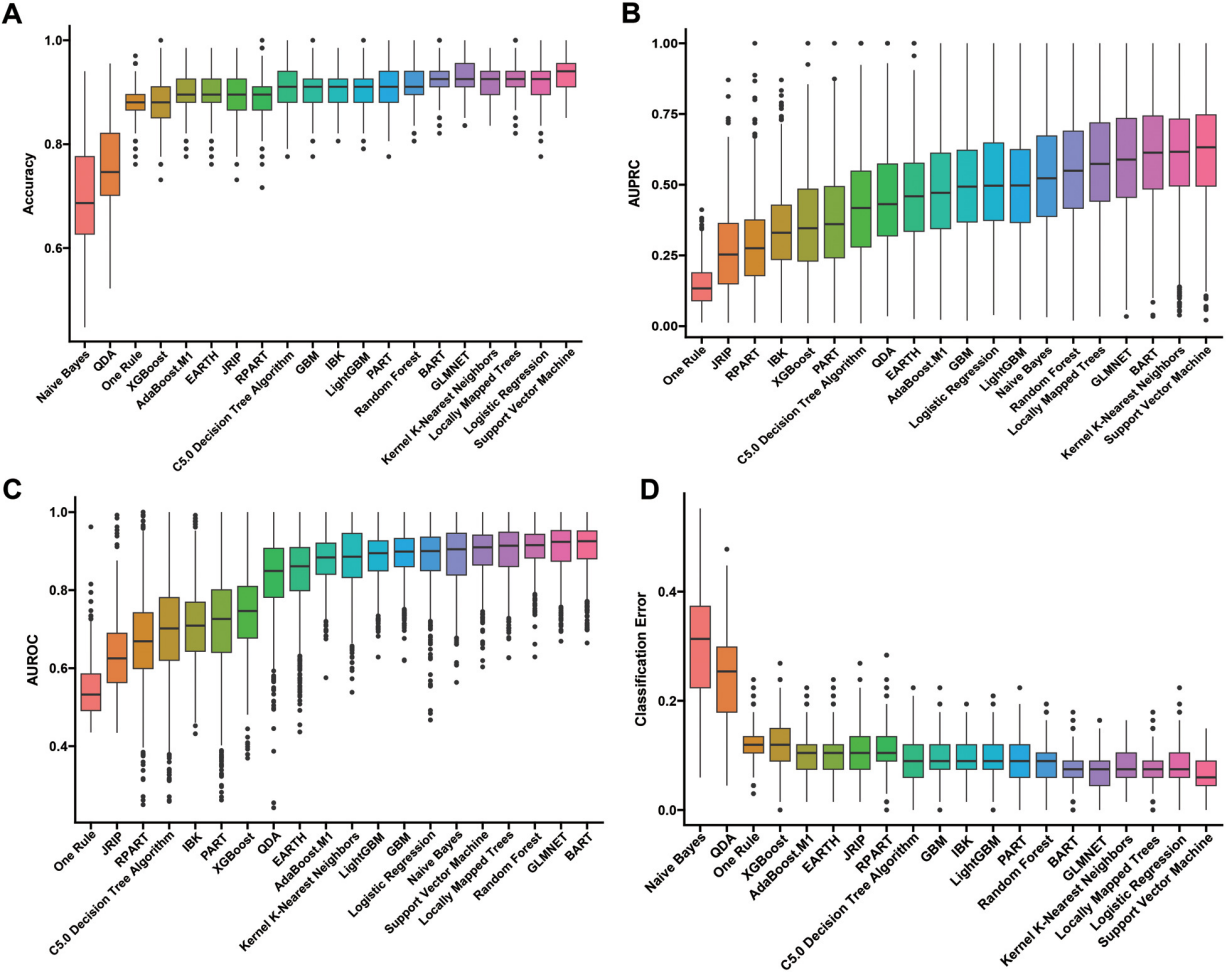
Performance comparison of 20 machine learning algorithms using various metrics. **(A-D)**. The results of evaluation methods such as accuracy, AUPRC, AUROC, and classification error. QDA, quadratic discriminant analysis; EARTH, extremely randomized trees; RPART, recursive partitioning and regression trees; GBM, gradient boosting machine; IBK, instance-based k-NN; LightGBM, light gradient boosting machine; PART, partial least squares regression; BART, Bayesian additive regression trees; AUPRC, area under the precision-recall curve; AUROC, area under the receiver operating characteristic curve.

**Figure 4:**
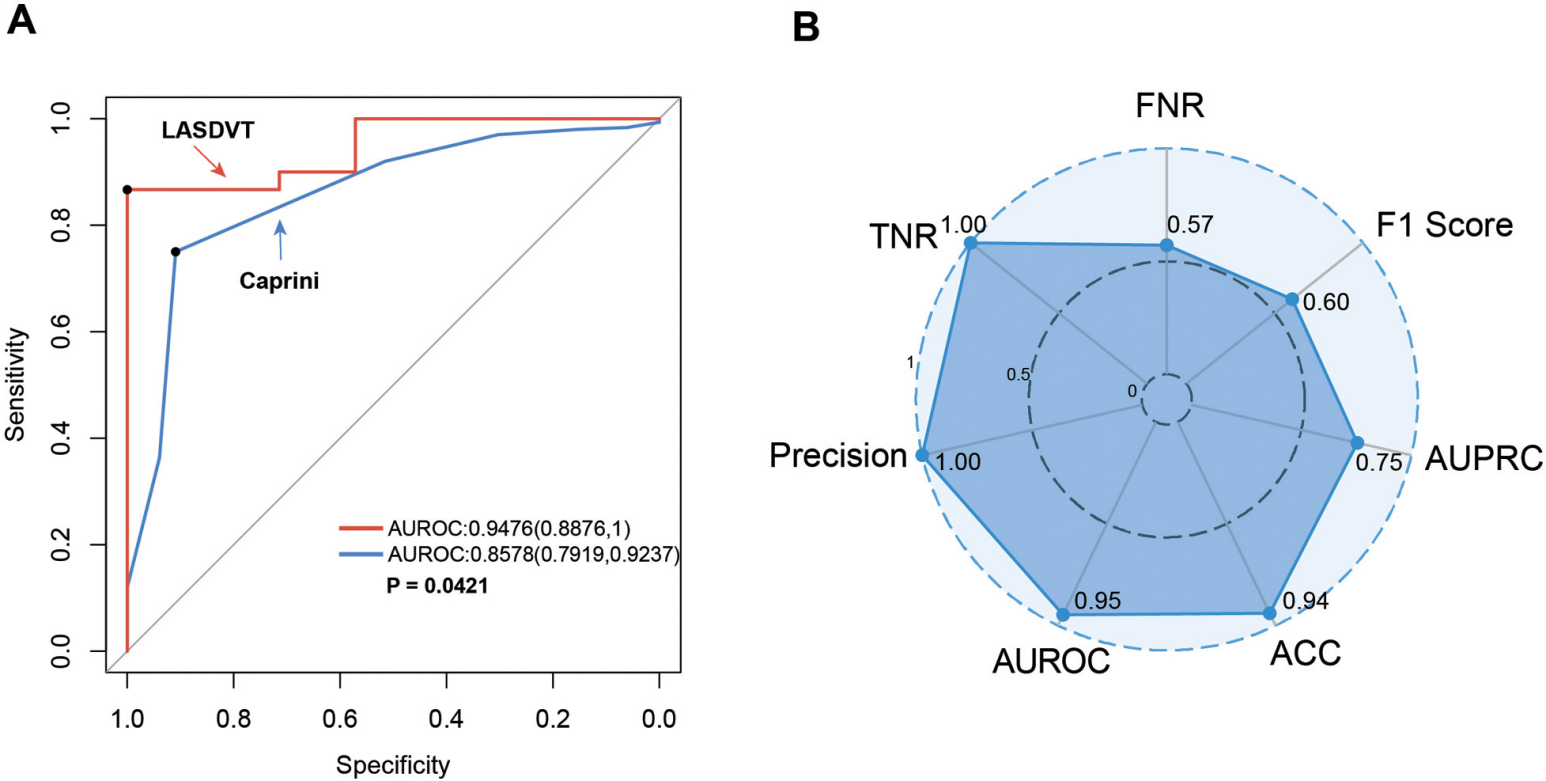
Performance demonstration of the LASDVT model. **(A)** The results of the comparison of the ROC curves for both the Caprini score and the LASDVT model. **(B)** Scoring results of the LASDVT model under several evaluation metrics. LASDVT, laparoscopic abdominal surgery deep vein thrombosis model; AUROC, area under the receiver operating characteristic curve; TNR, true negative rate; FNR, false negative rate; AUPRC, area under the precision-recall curve; ACC, accuracy; AUROC, area under the receiver operating characteristic curve.

**Figure 5:**
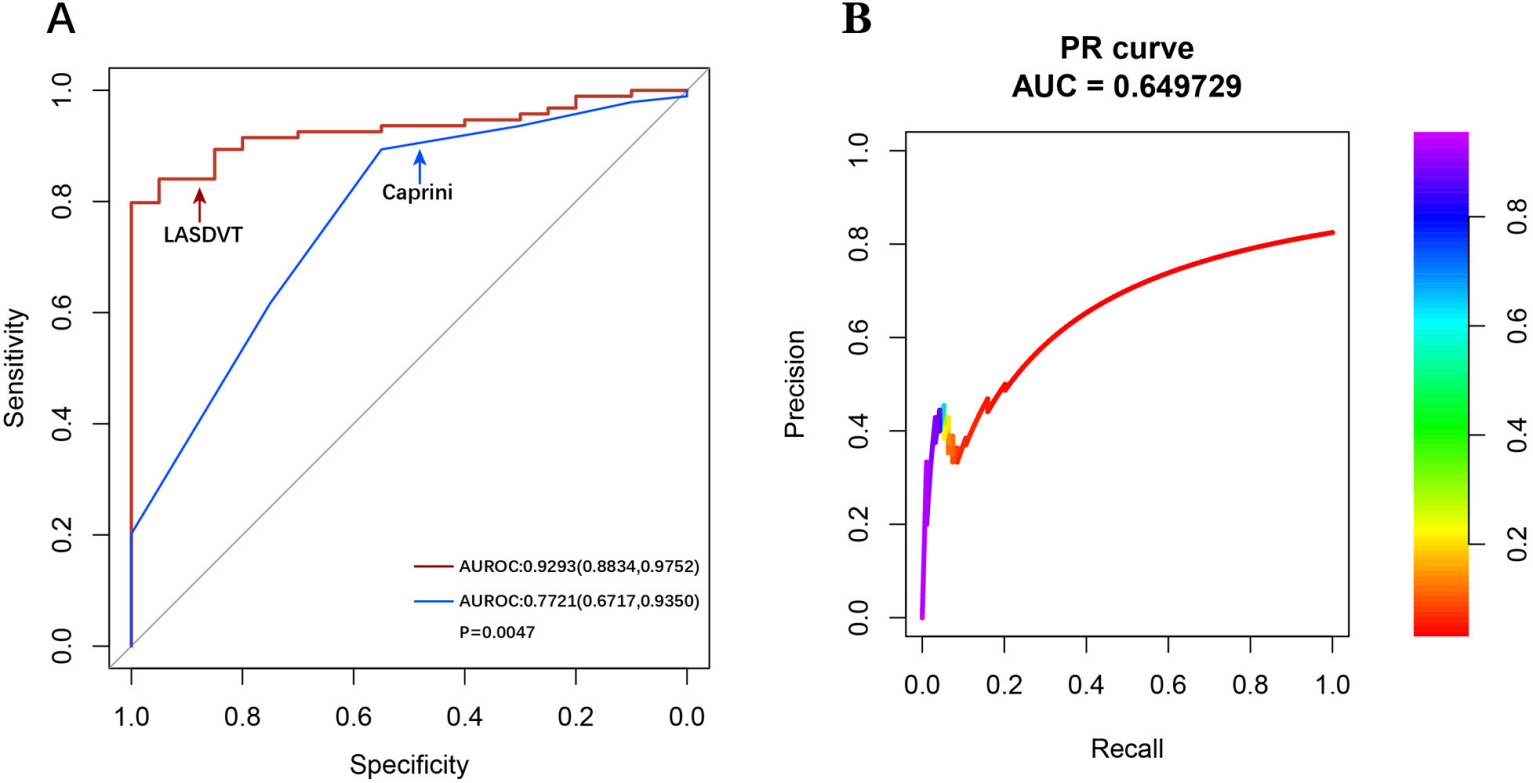
Performance demonstration of the LASDVT model. **(A)** The results of the comparison of the ROC curves for both the Caprini score and the LASDVT model. **(B)** the AUPRC of validation set for the LASDVT model. AUPRC, area under the precision-recall curve; ROC, area under the receiver operating characteristic curve.

Our construction for the ML model is based on the mlr3 package for R and uses 20 algorithms (including recursive partitioning and regression trees (RPART), random forest, AdaBoost. M1, C5.0 Decision Tree Algorithm, Instance-Based k-NN (IBK), XGBoost, Quadratic Discriminant Analysis (QDA), Naive Bayes, Logistic Regression, GLMNET, JRIP, (LMT), Bayesian Additive Regression Trees (BART), Kernel K-Nearest Neighbors (KKNN), Support Vector Machine (SVM), Light Gradient Boosting Machine (LightGBM), One Rule (OneR), Partial Least Squares Locally Mapped Trees Regression (PART), Extremely Randomized Trees (EARTH), Gradient Boosting Machine (GBM) to benchmark the meaningful features extracted above. To evaluate model performance, we employed a comprehensive suite of ML metrics, including the ACC, AUROC, AUPRC, CE, F1 score, FDR, FNR, FPR, TPR, and TNR (Table 3). Our evaluation metrics, including ACC, AUPRC, and AUROC, indicate that algorithms such as BART, GLMNET, KKNN, LMT, logistic regression, and SVM achieve higher accuracy. Conversely, naive Bayes and QDA performed less favorably in terms of accuracy (Figures 3A–C). Algorithms such as BART, GLMNET, KKNN, LMT, logistic regression, and SVM demonstrated high accuracy (low classification error rate) when evaluated using the CE metric (Figure 3D). From an initial evaluation of 20 algorithms, we narrowed the selection to the five with the strongest overall performance: BART, GLMNET, KKNN, LMT, and SVM. We then employed the stacking method to create the final model. The final stacked model had a value of 0.6154 for sensitivity and a value of 0.9917 for specificity. The model had good predictive value for the occurrence of deep vein thrombosis in patients after laparoscopy, with an AUROC of 0.9476, which was better than that of the Caprini score (*p* = 0.0421; Figure 4). Meanwhile, we additionally selected 114 patients for external validation, and the results showed that the ROC of validation set for the LASDVT model was 0.9293 and the AUPRC was 0.6497. The effect of the LASDVT model was statistically different (delong test, *p* = 0.0047; Figure 5) and superior to the Caprini score. Our results show that our model shows good generalization ability and robustness both in internal and external data.

**Table 3:** Benchmarking of 20 algorithms.

Learner	ACC	AUROC	AUPRC	CE	F1 Score	FDR	FNR	FPR	Precision	Recall	TNR
RPART	0.888 ± 0.039	0.665 ± 0.114	0.288 ± 0.145	0.112 ± 0.039	0.33 ± 0.176	0.56 ± 0.275	0.708 ± 0.193	0.047 ± 0.037	0.44 ± 0.275	0.292 ± 0.193	0.953 ± 0.037
Random forest	0.913 ± 0.031	0.909 ± 0.048	0.551 ± 0.184	0.087 ± 0.031	0.356 ± 0.173	0.307 ± 0.302	0.764 ± 0.17	0.012 ± 0.014	0.693 ± 0.302	0.236 ± 0.17	0.988 ± 0.014
AdaBoost.M1	0.901 ± 0.033	0.877 ± 0.061	0.478 ± 0.183	0.099 ± 0.033	0.384 ± 0.179	0.474 ± 0.27	0.663 ± 0.205	0.036 ± 0.027	0.526 ± 0.27	0.337 ± 0.205	0.964 ± 0.027
C5.0 decision tree Algorithm	0.91 ± 0.037	0.692 ± 0.128	0.418 ± 0.186	0.09 ± 0.037	0.434 ± 0.188	0.378 ± 0.301	0.632 ± 0.194	0.031 ± 0.031	0.622 ± 0.301	0.368 ± 0.194	0.969 ± 0.031
IBK	0.902 ± 0.03	0.709 ± 0.097	0.338 ± 0.148	0.098 ± 0.03	0.466 ± 0.166	0.49 ± 0.209	0.531 ± 0.195	0.05 ± 0.027	0.51 ± 0.209	0.469 ± 0.195	0.95 ± 0.027
XGBoost	0.883 ± 0.039	0.743 ± 0.102	0.359 ± 0.169	0.117 ± 0.039	0.324 ± 0.171	0.588 ± 0.255	0.697 ± 0.184	0.054 ± 0.037	0.412 ± 0.255	0.303 ± 0.184	0.946 ± 0.037
QDA	0.758 ± 0.077	0.833 ± 0.098	0.449 ± 0.178	0.242 ± 0.077	0.381 ± 0.121	0.728 ± 0.118	0.24 ± 0.199	0.24 ± 0.092	0.272 ± 0.118	0.76 ± 0.199	0.76 ± 0.092
Naive Bayes	0.696 ± 0.098	0.889 ± 0.074	0.535 ± 0.2	0.304 ± 0.098	0.373 ± 0.124	0.754 ± 0.104	0.112 ± 0.134	0.325 ± 0.11	0.246 ± 0.104	0.888 ± 0.134	0.675 ± 0.11
Logistic regression	0.917 ± 0.033	0.885 ± 0.075	0.508 ± 0.19	0.083 ± 0.033	0.508 ± 0.178	0.376 ± 0.238	0.532 ± 0.204	0.033 ± 0.026	0.624 ± 0.238	0.468 ± 0.204	0.967 ± 0.026
GLMNET	0.927 ± 0.029	0.908 ± 0.061	0.589 ± 0.192	0.073 ± 0.029	0.509 ± 0.175	0.222 ± 0.23	0.594 ± 0.191	0.014 ± 0.016	0.778 ± 0.23	0.406 ± 0.191	0.986 ± 0.016
JRIP	0.892 ± 0.039	0.63 ± 0.094	0.268 ± 0.148	0.108 ± 0.039	0.349 ± 0.174	0.518 ± 0.288	0.697 ± 0.194	0.044 ± 0.037	0.482 ± 0.288	0.303 ± 0.194	0.956 ± 0.037
LMT	0.925 ± 0.03	0.899 ± 0.066	0.573 ± 0.191	0.075 ± 0.03	0.504 ± 0.177	0.26 ± 0.241	0.592 ± 0.194	0.018 ± 0.019	0.74 ± 0.241	0.408 ± 0.194	0.982 ± 0.019
BART	0.922 ± 0.03	0.91 ± 0.06	0.603 ± 0.185	0.078 ± 0.03	0.431 ± 0.173	0.172 ± 0.24	0.707 ± 0.179	0.008 ± 0.01	0.828 ± 0.24	0.293 ± 0.179	0.992 ± 0.01
KKNN	0.92 ± 0.029	0.88 ± 0.078	0.6 ± 0.18	0.08 ± 0.029	0.504 ± 0.173	0.348 ± 0.241	0.559 ± 0.188	0.027 ± 0.021	0.652 ± 0.241	0.441 ± 0.188	0.973 ± 0.021
SVM	0.933 ± 0.027	0.899 ± 0.06	0.611 ± 0.184	0.067 ± 0.027	0.54 ± 0.174	0.164 ± 0.216	0.583 ± 0.192	0.01 ± 0.014	0.836 ± 0.216	0.417 ± 0.192	0.99 ± 0.014
LightGBM	0.906 ± 0.032	0.884 ± 0.058	0.495 ± 0.177	0.094 ± 0.032	0.39 ± 0.182	0.433 ± 0.278	0.675 ± 0.19	0.029 ± 0.024	0.567 ± 0.278	0.325 ± 0.19	0.971 ± 0.024
One rule	0.879 ± 0.032	0.544 ± 0.068	0.145 ± 0.073	0.121 ± 0.032	0.157 ± 0.15	0.776 ± 0.226	0.874 ± 0.146	0.038 ± 0.026	0.224 ± 0.226	0.126 ± 0.146	0.962 ± 0.026
PART	0.909 ± 0.037	0.712 ± 0.131	0.371 ± 0.178	0.091 ± 0.037	0.435 ± 0.187	0.386 ± 0.295	0.628 ± 0.194	0.032 ± 0.032	0.614 ± 0.295	0.372 ± 0.194	0.968 ± 0.032
EARTH	0.898 ± 0.034	0.842 ± 0.093	0.454 ± 0.169	0.102 ± 0.034	0.407 ± 0.175	0.506 ± 0.236	0.615 ± 0.201	0.044 ± 0.028	0.494 ± 0.236	0.385 ± 0.201	0.956 ± 0.028
GBM	0.907 ± 0.033	0.891 ± 0.058	0.494 ± 0.177	0.093 ± 0.033	0.396 ± 0.181	0.434 ± 0.276	0.667 ± 0.192	0.029 ± 0.023	0.566 ± 0.276	0.333 ± 0.192	0.971 ± 0.023

RPART, recursive partitioning and regression trees, IBK, instance-based k-NN; QDA, quadratic discriminant analysis; LMT, locally mapped trees; BART, Bayesian additive regression trees; KKNN, kernel K-nearest neighbors; SVM, support vector machine; LightGBM, light gradient boosting machine; OneR, one rule; PART, partial least squares regression; EARTH, extremely randomized trees; GBM, gradient boosting machine. ACC, accuracy; AUROC, area under the receiver operating characteristic curve; AUPRC, area under the precision-recall curve; CE, classification error; FDR, false discovery rate; FNR, false negative rate; FPR, false positive rate; TNR, true negative rate.

### Interpretation of the model

#### Global interpretation

**Figure 6:**
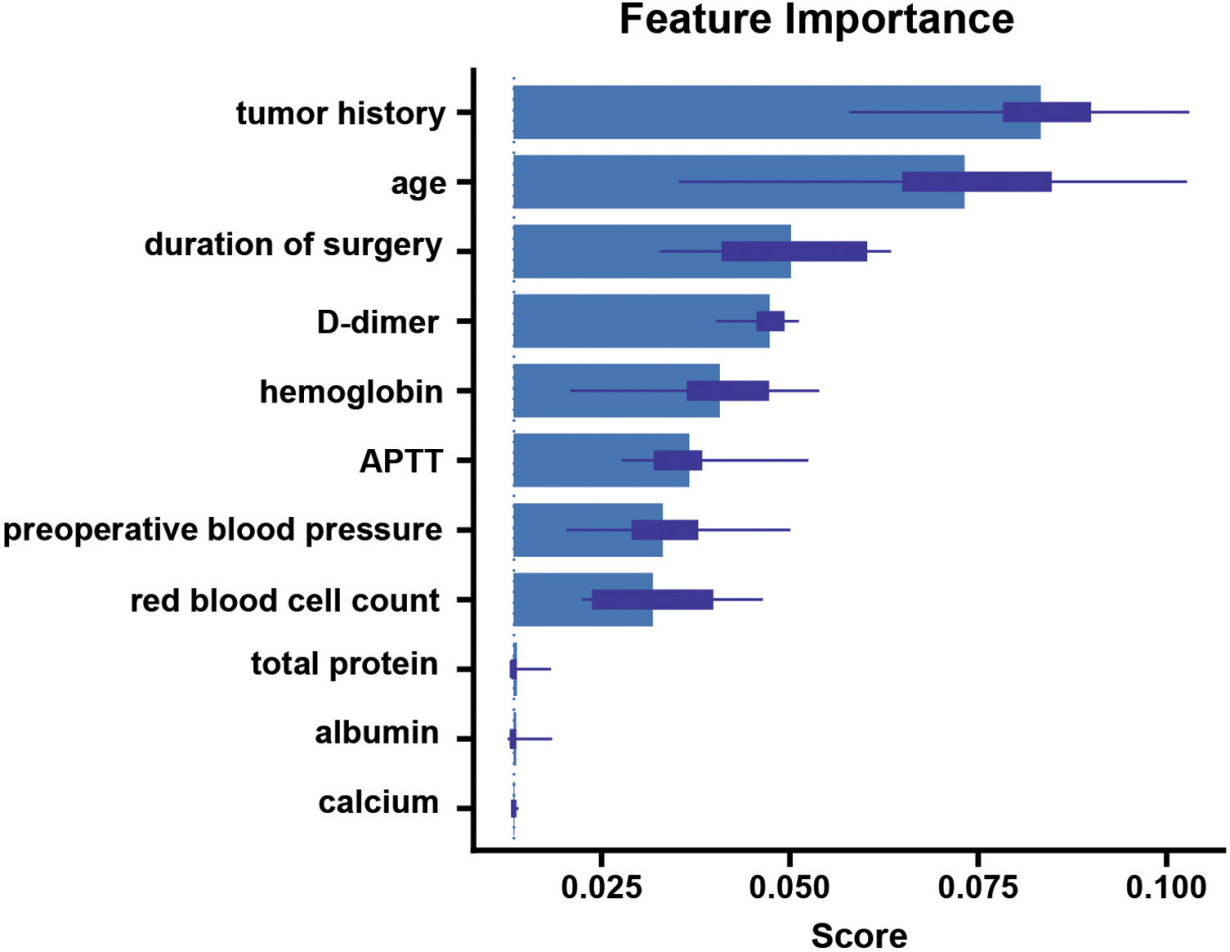
A global interpretation of the LASDVT model. The five most important features in the LASDVT model were tumor history, age, time to surgery, D-dimer level, and hemoglobin level. APTT: activated partial thromboplastin time.

This refers to understanding the overall behavior of the model and its predictions across the entire dataset. According to our model, the most important features influencing the predictions, ranked by their importance, were tumor history, age, time to surgery, D-dimer level, and hemoglobin level (Figure 6).

#### Local interpretation

**Figure 7:**
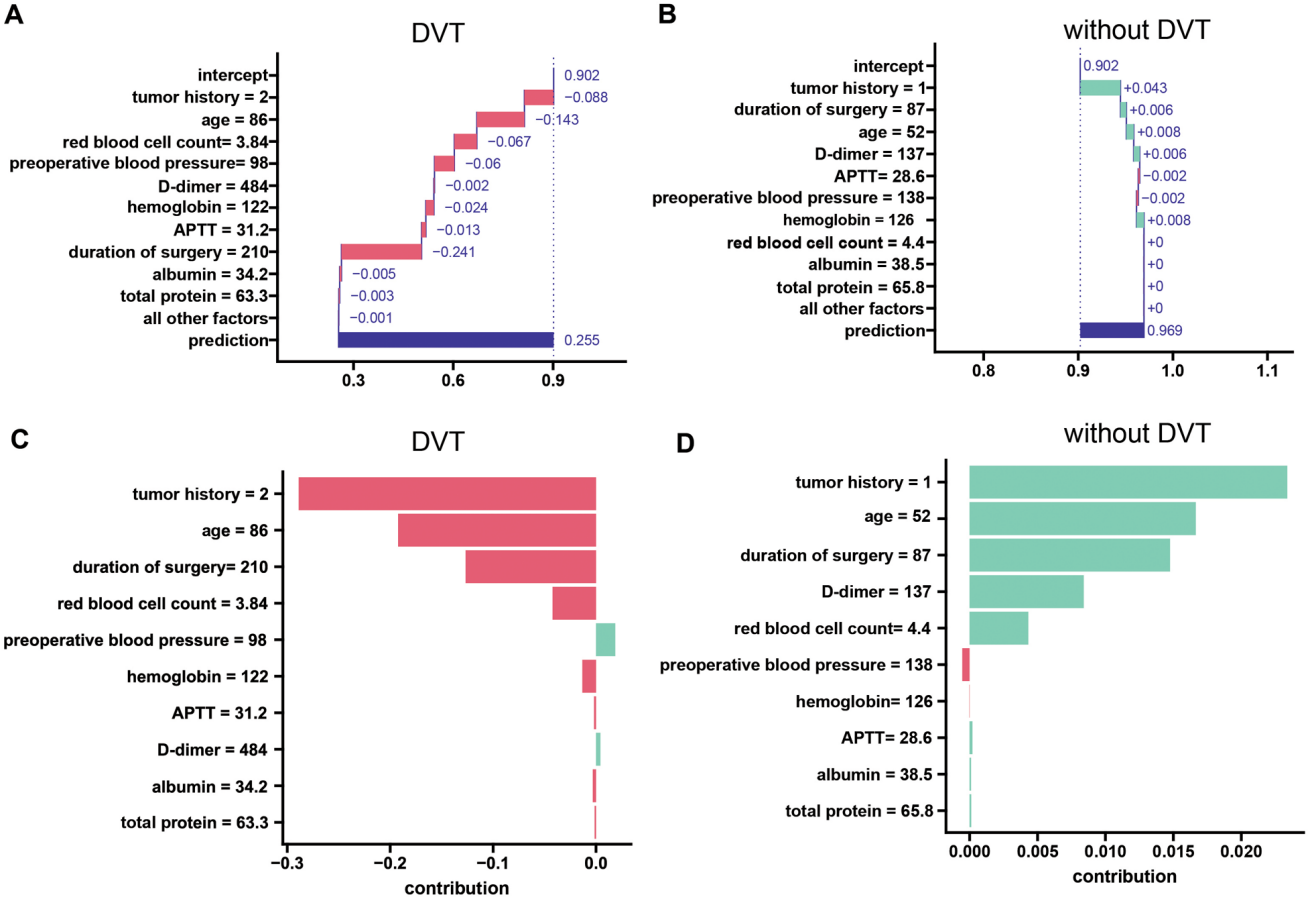
A local interpretation of the LASDVT model. **(A)** Decompositional interpretation of factors that can predict the development of deep vein thrombosis in patients after laparoscopy. **(B)** Decomposition interpretation of factors that can exclude the possibility of deep vein thrombosis in patients after laparoscopy. **(C)** Shapley additive interpretation of factors that can predict the development of deep vein thrombosis in patients after laparoscopy. **(D)** Shapley additive interpretation of factors that can rule out the possibility of deep vein thrombosis in patients after laparoscopy. APTT, activated partial thromboplastin time. DVT, deep venous thrombosis.

Refers to understanding the contribution of individual features to the model's predictions for a specific instance (sample). We randomly selected two patients (one who developed DVT after laparoscopy and the other who did not develop DVT after laparoscopy). We applied two common localized interpretation methods—decomposition interpretation and Shapley additive explanation (SHAP)—to each of the two patients' data. The results are as follows:

The breakdown plot (Figures 7A,B) deconstructs the model's predictions by visualizing the influence of each variable and its relative contribution. It achieves this by assigning an importance score to each variable, with the sum of these scores closely approximating the overall prediction. The breakdown charts visualize the contribution of each variable to the prediction. In this study, we investigated the relationships between various factors, including age, tumor history, hemoglobin, red blood cell count, preoperative blood pressure, duration of surgery, APTT, D-dimer, total protein, albumin, and calcium, and the likelihood of developing DVT after laparoscopy. We employed decomposition analysis to interpret these relationships, and the results are presented in a clear and informative breakdown chart. In patients who developed deep vein thrombosis after laparoscopy, study variables such as tumor history, age, red blood cell count, blood pressure, D-dimer, hemoglobin, APTT, duration of surgery, albumin, and total protein were predictive of the development of deep vein thrombosis after laparoscopy. The three strongest predictors were time to surgery, patient age, and tumor history. In this patient who did not develop DVT after laparoscopy, the 3 study variables that had a better predictive effect for excluding the development of DVT after laparoscopy were tumor history, hemoglobin, and age.

SHAP leverages Shapley values, a game theory concept, to quantify the individual contribution of each feature to a model's predictions. We applied Shapley's method of additive interpretation to study variables such as age, tumor history, hemoglobin, red blood cell count, preoperative blood pressure, duration of surgery, APTT, D-dimer, total protein, albumin, and calcium that can predict the likelihood of DVT in patients after laparoscopy and drew the following conclusions (Figures 7C,D). We found that three study variables, namely, tumor history, age, and duration of surgery, were effective in predicting and excluding the occurrence of deep vein thrombosis after laparoscopy.

## Discussion

Artificial Intelligence (AI) is rapidly transforming healthcare, revolutionizing traditional practices. AI applications in medicine not only enhance diagnostic accuracy but also significantly improve healthcare efficiency and provide a superior patient experience. Chronic complications of DVT include post thrombotic syndrome (25%-38%) and venous ulceration (9.8%). Pulmonary embolism (6%-32%), a more acute complication, can be fatal in 5%-10% of patients. Less common complications include chronic thromboembolic pulmonary hypertension, sudden death, and limb loss (22). Early and accurate prediction of DVT is critical because it can significantly impact patients' quality of life and even pose a life-threatening risk. Currently, the diagnosis and prediction of LEDVT primarily rely on scoring systems such as the Caprini and Padua scores, along with laboratory tests and ultrasonography (18). However, their predictive effectiveness in laparoscopic surgery remains uncertain (19, 20).

Artificial Intelligence (AI) encompasses a variety of technologies that share the common goal of computationally simulating human intelligence. Machine Learning (ML) is a branch of AI that focuses on making predictions by using mathematical algorithms to identify patterns in data (23). ML is a rapidly growing field in medicine, with significant research efforts focused on applying its capabilities to address medical challenges (24). ML in medicine can lead to more accurate diagnostic algorithms and personalized patient care (25, 26). Stacked ensemble methods are a type of ML approach that combines multiple classifier models in a hierarchical structure (27, 28). In ML, stacking can be employed as an ensemble method to address both model errors and dataset biases (29). Stacked integrated learning (SEL) is an algorithmic structure that consists of multiple levels of ML algorithms (27, 30). This type of ML produces more reliable models (29).

The model had good predictive value for the occurrence of DVT in patients after laparoscopy, with an AUROC of 0.9476. The model had good composite performance. This model outperformed the Caprini score in predicting the incidence of deep vein thrombosis. The model we constructed demonstrates a higher probability of accurately predicting or excluding the risk of postoperative VTE in patients undergoing abdominal surgery, thus offering a potential improvement over existing VTE prediction tools. Lee Hwangbo (29) et al. developed a mortality prediction model for acute ischemic stroke patients who did not receive reperfusion therapy; this model used a stacked integrated learning model with an AUROC of 0.783 for 6-month mortality prediction, which is a better supplement to the clinical methods for predicting mortality in acute ischemic stroke patients. The ML models developed by Tao Yu (31) et al. accurately predict post thrombotic syndrome (PTS). This approach demonstrates strong predictive ability and generalizability, potentially allowing clinicians to better select patients for endovascular surgery. Hua Liu (32) et al. built and externally validated a novel ML model to predict venous thromboembolism (VTE) in hospitalized young and middle-aged patients. Five algorithms (logistic regression, decision tree, feed-forward neural network, support vector machine, and random forest) were employed to train and refine the model. The support vector machine model performed the best, with 95% CI AUC values of 0.806∼0.944, a sensitivity of 59%, a specificity of 99%, and an accuracy of 87%. The risk of venous thromboembolism can be accurately predicted using a support vector machine model with a clinical dataset of young and middle-aged hospitalized patients. Our model leverages a stacking approach, screening the top 5 basic models from 20 candidates for integration. This integrated model can effectively predict the risk of DVT after laparoscopic surgery for abdominal surgery and can serve as a good guide to the clinic. Our study revealed that patients who developed DVT after laparoscopic surgery were older, on average, than those who remained DVT-free postoperatively. This age difference may be attributed to factors such as increased vascular intima roughness, intimal damage, procoagulant production, and a greater incidence of comorbidities such as cardiovascular disease and tumors in older individuals (33). In addition, patients with malignant tumors often exhibit low fibrinolytic capacity and hyperfibrinogenemia. Tumors may secrete both procoagulant substances, which promote platelet aggregation and release, and inhibitors of fibrinolytic activity, leading to a hypercoagulable state in the body. Certain chemotherapeutic drugs can cause deficiencies in protein C, protein S, and antithrombin, increasing the risk of blood clots (deep vein thrombosis or DVT). Additionally, compression of blood vessels by tumors, prolonged bed rest, and other factors can also promote DVT formation (34). Our findings also revealed that a higher percentage of patients in the postoperative DVT group than in the group without postoperative DVT had a history of tumors. Patients who developed postoperative DVT had higher preoperative blood pressure readings compared to those who did not develop DVT. Previous studies have shown that high blood pressure leads to faster blood flow and increased shear stress, which damages venous endothelial cells and increases the likelihood of releasing procoagulant factors; high blood pressure leads to increased blood viscosity, red blood cell aggregation, and platelet activation, which increase the risk of thrombosis. Patients who developed postoperative DVT underwent significantly longer surgeries compared to those who did not develop DVT. While laparoscopic surgery offers a significant advantage over open surgery in terms of reduced operative time, patients are still required to remain in a supine or sitting position for extended periods. This can lead to slower blood flow in the lower extremities, increasing the risk of blood stasis. Stasis, in turn, promotes platelet aggregation and thrombosis. Additionally, surgical trauma itself activates the coagulation cascade, further contributing to a hypercoagulable state. Furthermore, intraoperative vascular injury and prolonged use of carbon dioxide pneumoperitoneum during laparoscopy have been associated with an increased risk of DVT (35, 36). Our findings align with our existing DVT diagnostic approach, as D-dimer levels were significantly elevated in the postoperative DVT group compared to the control group, while APTT was conversely lower in the DVT group (37). Previous studies have rarely reported that both total protein and albumin levels are lower in patients who develop postoperative DVT compared to those who do not. This finding might be partially explained by the link between hypoalbuminemia and decreased colloid osmotic pressure. This pressure drop leads to fluid movement from the intravascular space to the extravascular space, causing tissue edema. Consequently, it may contribute to blood flow stasis and heighten the risk of DVT. Although our model outperforms the widely used Caprini score in clinical practice, it still has several limitations. First, this was a single-center study at Wenzhou Central Hospital, limiting the generalizability of the findings to other institutions. Future studies should involve multiple centers for a more representative sample. Second, the sample size was relatively small, which might impact certain statistical analyses and the generalizability of the results. Third, the study focused on several abdominal surgery-related diseases, limiting the generalizability of the findings to other disease types. While this focus provided a more targeted investigation, it is essential to acknowledge this limitation in future applications. We collected and reviewed clinical charts and related data of patients who underwent laparoscopic surgery from January 3, 2023, to December 19, 2023, in the hepatobiliary surgery department at our hospital. This is only the case data of hepatobiliary surgery in general surgery in our hospital, but it basically covers the basic diseases in general abdominal surgery, and we have performed external validation of the model. We will incorporate more samples and even multicenter data to further improve our model in order to translate the results to better serve research and clinical work.

Fourth, the model relied solely on clinical data, including laboratory tests and vital signs, neglecting other potentially contributing factors, such as environmental and genetic elements. Fifth, the marked disparity in sample sizes between the observation group (postoperative DVT, *n* = 33) and control group (non-DVT, *n* = 302) indeed constitutes a critical methodological consideration. While we implemented ensemble learning techniques (e.g., stacked generalization) to enhance model robustness, we fully acknowledge that the inherent class imbalance may have introduced predictive bias toward the majority class, potentially impacting sensitivity metrics for DVT detection.

Notably, our external validation cohort (*n* = 114) demonstrated preserved discriminative capacity (AUROC = 0.9293), suggesting reasonable generalizability within the current sample constraints. However, we concur that the limited representation of DVT-positive cases and the absence of explicit balancing strategies (e.g., SMOTE, class-weighted learning) represent study limitations. These factors may affect model performance in clinical scenarios with higher DVT prevalence. Future studies could further enhance the clinical application value of the model by introducing more advanced balancing techniques or expanding the multicenter sample size, especially by increasing the proportion of DVT-positive cases. Additionally, as a retrospective study, the selection of cases and controls might introduce selection bias.

## Conclusion

This study constructed an integrated ML model that utilizes both preoperative and intraoperative data to predict the occurrence of DVT in patients undergoing laparoscopic abdominal surgery. Our model outperforms the Caprini score in predicting DVT occurrence. Future validation in larger centers and diverse populations can establish its broader application, allowing healthcare professionals to more effectively identify potential DVT cases and improve patient outcomes. Subsequent translation of the results can be further enhanced by interdisciplinary collaboration, such as with computer scientists, to develop specialized software that optimizes clinical diagnosis and treatment.

## Data Availability

The raw data supporting the conclusions of this article will be made available by the authors, without undue reservation.

## Supplementary material

Supplementary Table S1 Type of surgery and its percentage for 335 patients.

Supplementary Table S2 Indications for surgery and their percentage in 335 patients.

Supplementary Table S3 Baseline information for 114 patients.
