# Supplementary material for: A machine learning-based predictive model for the occurrence of lower extremity deep vein thrombosis after laparoscopic surgery in abdominal surgery
Source: Front Surg. 2025 May 30;12:1502944. doi: 10.3389/fsurg.2025.1502944 (PMC12162540; doi:10.3389/fsurg.2025.1502944)
Supplement: Supplementary Table S3 — Baseline information for 114 patients. [file Table3.docx]

**Table S3** Baseline information for 114 patients.

| **Characteristics** | **Parameter(n=114)** |
| --- | --- |
| Age(years) | 51.272±17.642 |
| Gender |  |
| Male | 52(45.61%) |
| Female | 62(54.39%) |
| Tumor history |  |
| Yes | 18(15.79%) |
| No | 96(84.21%) |
| Prothrombin percentage activity(%) | 106.487±14.262 |
| Hemoglobin(g/L) | 130.149±22.846 |
| Red blood cell count(10^12^/L) | 4.378±0.753 |
| Body weight(kg) | 60(55,70) |
| Height(cm) | 162(157.25,168.75) |
| Preoperative blood pressure(mmHg) | 132(117,145) |
| Duration of surgery(min) | 70(55,120) |
| Prothrombin time(s) | 11 (10.5,11.6) |
| Fibrinogen(g/L) | 3.28(2.94,3.79) |
| Thrombin time(s) | 15.2(14.3,15.9) |
| Activated partial thromboplastin time(s) | 30.05(28.13,31.88) |
| D-dimer(µg/L) | 137(137,159.25) |
| White blood cell count(10^9^/L) | 5.9(5.2,7.2) |
| Absolute neutrophil count(10^9^/L) | 3.74(2.89,5.11) |
| Absolute lymphocyte count(10^9^/L) | 1.88(1.5,2.64) |
| mean corpuscular hemoglobin (pg) | 29.7(28.3,31) |
| Platelet count(10^9^/L) | 228(193,262) |
| Total protein(g/L) | 68.1(64,71.4) |
| Albumin(g/L) | 41.65(38.78,44.5) |
| Globulin(g/L) | 25.95(23.3,28.1) |
| Total bilirubin(µmol/L) | 13.5(9.8,19.9) |
| Direct bilirubin(µmol/L) | 3.2(2.1,4.93) |
| Indirect bilirubin(µmol/L) | 9.9(7,12.525) |
| Glucose(mmol/L) | 5.2(4.6,6.2) |
| Urea(mmol/L) | 4.4(3.7,5.4) |
| Calcium(mmol/L) | 2.27(2.19,2.32) |
| C-reactive protein(mg/L) | 1.8(0.8,4.15) |
| DVT |  |
| Yes | 20(17.5%) |
| No | 94(82.46%) |
